# Characterization of Phyllobacterium spp. isolated from root nodules of Melilotus albus (white sweet clover) grown in Canada and description of Phyllobacterium meliloti sp. nov.

**DOI:** 10.1099/ijsem.0.006876

**Published:** 2025-08-07

**Authors:** Eden S. P. Bromfield, Sylvie Cloutier, Michael F. Hynes

**Affiliations:** 1 Agriculture and Agri-Food Canada, 960 Carling Ave., Ottawa, K1A 0C6, Canada; 2 Department of Biological Sciences, University of Calgary, Calgary, AB, Canada

**Keywords:** Canada, glyphosate, *Phyllobacterium meliloti *sp. nov., phylogenomics, prophage

## Abstract

Two novel bacterial strains isolated from root nodules of white sweet clover (*Melilotus albus*) plants grown at a Canadian site were previously characterized and placed in the genus *Phyllobacterium*. Here, we present phylogenomic and phenotypic data to support the description of strain T1293^T^ as representative of a novel species and present the first complete closed genome sequence of a bacterial strain (T1018) representing the species ‘*Phyllobacterium pellucidum*’. Phylogenetic analysis of genome sequences, as well as analysis of 53 core genes, placed novel strain T1293^T^ in a highly supported cluster of strains distinct from named *Phyllobacterium* species with *Phyllobacterium myrsinacearum* and *Phyllobacterium calauticae* as closest relatives. The highest average nucleotide identity and digital DNA–DNA hybridization values of genome sequences of T1293^T^ compared to closest species type strains (84.1 and 26.5%, respectively) are well below the threshold values for bacterial species circumscription. The genome of strain T1293^T^ has a size of 5,074,034 bp with a DNA G+C content of 55.1 mol% and possesses three plasmids with sizes of 397,619 bp, 476,847 bp and 519,835 bp. Detected in the genome were type III and type VI secretion system genes, implicated in plant–microbe and microbe–microbe interactions, but key nodulation, nitrogen-fixation and photosystem genes were not detected. A novel prophage (size ~41.5 kb) was also detected in the genome of T1293^T^. Tests using defined culture media revealed that novel strains T1293^T^ and T1018 were highly resistant to, and able to metabolize glyphosate, a widely used herbicide that has negative consequences for the environment and human health. Data for multiple morphological, physiological, biochemical and plant tests complemented the sequence-based data. The data presented support the description of a new species, and the name *Phyllobacterium meliloti* sp. nov. is proposed with T1293^T^=LMG 32641^T^=HAMBI 3765^T^ as the species type strain.

## Introduction

The bacterial genus *Phyllobacterium*, placed in the family *Bartonellaceae* [1], was originally named after bacteria isolated from leaf nodules of tropical ornamental plants [2]. Sixteen *Phyllobacterium* species have been described to date [3] of which 14 have validly published names. Most of these species are either free-living or associated with plants; only two species (*Phyllobacterium sophorae* [4] and *Phyllobacterium trifolii* [5]) are able to form a symbiotic association with a plant host.

In a previous study [6], bacteria were isolated from root nodules of *Melilotus albus* (white sweet clover) grown at a field site in Canada that had no history of cultivation. Phylogenetic analysis of four core gene sequences placed these bacterial isolates in several distinct lineages that were assigned to the genera *Ensifer*, *Rhizobium* and *Phyllobacterium* [7]. Two of these lineages, represented by strains T1293^T^ and T1018, were placed in the genus *Phyllobacterium* and shown to be novel genospecies. Since that time, a strain designated BT25^T^, closely related to strain T1018, was isolated from soil in Korea and described as a species named ‘*Phyllobacterium pellucidum*’ [8].

In the present work, we carried out detailed phylogenetic, genomic and phenotypic analyses of novel strain T1293^T^ and sequence-based analyses of strain T1018. Based on the data generated, we propose a novel species named *Phyllobacterium meliloti* sp. nov. with T1293^T^ as the type strain and present the first complete closed genome sequence of a bacterial strain (T1018) representing the species ‘*P. pellucidum*’.

## Bacteria, habitat and isolation

Novel bacterial strains, *P. meliloti* sp. nov. T1293^T^ and ‘*P. pellucidum*’ T1018, were isolated in 1992 from root nodules of *M. albus* cultivar Polara plants grown at an uncultivated field site in Ottawa, Ontario, Canada, that had no history of agriculture [6]; the soil at this site was a sandy loam (pH 6.1, water) with good drainage. The bacterial isolation procedure was as follows: root nodules were washed and surface-sterilized [5 s immersion in 70% ethanol followed by 20 s exposure to sodium hypochlorite (14% available chlorine)]; nodules were then washed in water for 10 min with frequent changes. Surface-sterilized nodules were crushed, and a droplet of the contents streaked on nutrient agar medium; pure bacterial cultures were obtained by repeated streaking and single-colony picking [6].

*P. meliloti* sp. nov. T1293^T^ was deposited in the BCCM/LMG Bacteria Collection, University of Ghent, Belgium, as collection no. LMG 32641^T^ and in the HAMBI Microbial Culture Collection, University of Helsinki, Finland, as collection no. HAMBI 3765^T^.

Novel strain, ‘*P. pellucidum*’ T1018 was deposited in the BCCM/LMG Bacteria Collection, University of Ghent, Belgium, as LMG collection no. LMG 32375.

Bacterial reference strains used in phenotypic tests were as follows: *Phyllobacterium calauticae* R2-JL^T^ and *Phyllobacterium myrsinacearum* LMG 2t2^T^ from the BCCM/LMG Bacteria Collection, University of Ghent, Belgium; ‘*P. pellucidum*’ BT25^T^ from the Culture Collection Division, Biological Resource Center, National Institute of Technology and Evaluation (NBRC), Chiba, Japan; and a derivative of *Escherichia coli* K12 (QIAGEN EZ competent cells) provided in QIAGEN PCR Cloning plus Kits (https://www.qiagen.com/us).

## DNA sequencing, genomic and phylogenetic analyses

Genomic DNA was extracted and purified from bacterial cells grown for 3 days at 28 °C on modified yeast extract mannitol (YEM) agar medium having the following composition (g l^−1^): yeast extract (Thermo Scientific^™^ Oxoid^™^), 1.5; mannitol, 1.0; NaCl, 0.1; K_2_HPO_4_, 0.5; MgSO_4_· 7H_2_O, 0.2; bacteriological agar (Thermo Scientific^™^ Oxoid^™^), 18.0.

The complete genomes of novel strains T1293^T^ and T1018 were sequenced at the Genome Quebec Innovation Centre, Canada, using Pacific Biosciences (PacBio) Sequel Single-Molecule Real-Time technology as detailed previously [9]; Flye software (version 2.9) [10] was employed for sequence assembly.

Estimated genome coverage for novel strain T1293^T^ was 3,369-fold with 91,069 polymerase reads and an average read length of 123,841 bp; for strain T1018, coverage was 2,247-fold with 61,355 polymerase reads and an average read length of 93,837 bp. The complete closed genome sequences of strains T1293^T^ and T1018 were of high quality [11] with completeness values of 99.0% (one hundredth percentile) and 99.4% (one hundredth percentile) and low contamination values of 1.2 and 0.9%, respectively.

The complete closed genome sequence of novel strain T1293^T^ has a size of 5,074,034 bp with a DNA G+C content of 55.1 mol% (Table 1). Three circular plasmids possessing *repABC* genes encoding proteins involved in plasmid replication and segregation [12] were detected in this novel strain as follows: pT1293a (397,619 bp), pT1293b (476,847 bp) and pT1293c (519,835 bp). It is noteworthy that the detection of three plasmids in the genome of strain T1293^T^ is supported by the results of plasmid profile analysis (agarose gel electrophoresis) in our previous work [7].

**Table 1.** Characteristics of genome sequences of *Phyllobacterium meliloti* sp. nov. T1293^T^, ‘*Phyllobacterium pellucidum*’ T1018 and reference strains

Characteristic	*P. meliloti* sp. nov. T1293^T^	*Phyllobacterium* sp. AFS055582	*Phyllobacterium* sp. AFS073063	*Phyllobacterium* sp. P30BS-XVII	*P. myrsinacearum* LMG 2** t2^T^**	*P. calauticae* R2-JL^T^	‘*P. pellucidum*’ T1018	‘*P. pellucidum*’ BT25^T^
Isolation source	Root nodule [6]	Insect [18]	*Zea mays* root [18]	Soil [19]	Leaf nodule [2]	Sediment [43]	Root nodule [6]	Soil [8]
Genome assembly (no. of contigs) and accession no.	Complete (4) CP088273–CP088276	Draft (50) UEIC01	Draft (71) UCEP01	Draft (23) JACGXO01	Draft (17) SHLH01	Draft (45) JAGENB01	Complete (2) CP088292–CP088293	Draft (16) JABUMX01
Genome size (bp)	5,074,034	5,143,721	5,386,454	5,250,153	8,264,165	7,522,254	3,872,269	4,660,625
Plasmid no. (size, bp)	3 (397,619; 476,847; 519,835)	na	na	na	na	na	1 (388,677)	na
Genes (total)	4,860	4,942	5,177	5,096	5,222	5,163	3,737	4,535
CDSs (total)	4,795	4,845	5,047	4,952	5,132	5,100	3,681	4,481
G+C content (mol%)	55.1	54.7	55.0	55.1	59.5	59.1	59.1	59.1
No. of rRNA operons (5S, 23S, 16S)	3	1	1	1	1	1	2	1

na, not applicable.

The complete genome sequence of ‘*P. pellucidum*’ T1018 has a size of 3,872,269 bp with a DNA G+C content of 59.1 mol%. A single plasmid (pT1018 with a size of 388,677 bp) possessing *repABC* genes was detected in the genome of this strain (Table 1).

For phylogenetic analyses, nucleotide sequences were retrieved from genome sequences, and alignments were performed using muscle [13]. Phylogenetic analyses were done using MrBayes (software version 3.2.1) with default priors as described previously [14]. Best-fit substitution models were selected using ModelTest-NG [15] implemented in the web-based CIPRES Science Gateway version 3.3 [16]. In a previous study [17], we reported that the *recA* gene (encoding recombinase A involved in DNA repair) is a useful phylogenetic marker for rapid screening of bacterial isolates for potential novel genospecies. We performed BLASTn searches using the complete *recA* gene sequence of novel strain T1293^T^ (locus tag, LLE53_003560) as a query to locate sequences of any closely related strains in NCBI databases. Based on the results of these searches, we included the following unclassified but closely related strains (≥98.9% identity) to T1293^T^ in our phylogenetic and genomic analyses: AFS055582 and AFS073063 were isolated, respectively, from an insect and a root of a *Z. mays* (corn) plant [18], and strain P30BS-XVII was isolated from pastureland bulk soil [19]

In accordance with minimal standards proposed for the use of genome data for prokaryotic taxonomy [20], we verified that the 16S rRNA gene sequences of strains T1293^T^ and T1018 generated by the Sanger method [14] were identical to the respective 16S rRNA sequences generated by whole-genome sequencing.

The 16S rRNA gene is highly conserved in bacteria, and as such, its usefulness as a taxonomic tool is limited to assessment of genus level or higher taxonomic rank [20]. A Bayesian phylogenetic tree (Fig. S1, available in the online Supplementary Material) of nearly full-length 16S rRNA gene sequences (alignment length: 1,364 positions) of type strains of all 16 described *Phyllobacterium* species confirms placement of novel strains T1293^T^ and T1018 in the genus *Phyllobacterium*. Strain T1293 ^T^ is placed in a distinct cluster with the unclassified strains AFS055582, AFS073063 and P30BS-XVII that were retrieved from database searches. Placement in the 16S rRNA gene tree also indicates that the closest species relatives of T1293^T^ are type strains of *P. myrsinacearum* and *P. calauticae*, while the closest relative of T1018 is the type strain of ‘*P. pellucidum*’.

To further verify the taxonomic position of strains T1293^T^ and T1018, we carried out the following phylogenetic analyses employing strains AFS055582, AFS073063 and P30BS-XVII and 13 type strains of *Phyllobacterium* species as reference taxa: (1) MLSA of 53 concatenated single-copy core gene sequences encoding ribosome protein subunits (*rps*) [21] and (2) analysis of whole-genome sequences implemented in the Type Strain Genome Server (TYGS) [22]. Procedural details of these phylogenetic analyses were as described previously [23].

**Fig. 1.**
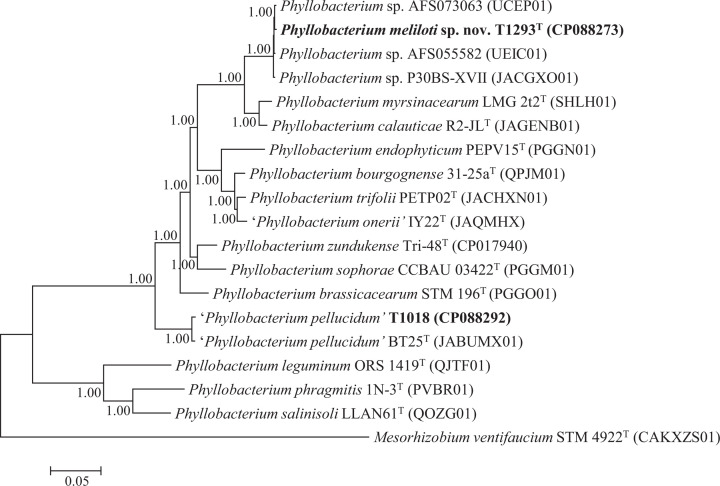
Bayesian phylogenetic tree (GTR+G+I substitution model) inferred from 53 full-length concatenated ribosome protein subunit (*rps*) gene sequences for *Phyllobacterium meliloti* sp. nov. strain T1293^T^ and reference taxa. Alignment length, 22,868 positions. NCBI sequence accession numbers are given in parentheses. *Mesorhizobium ventifaucium* STM 4922^T^ was used as the outgroup. Posterior probabilities ≥0.90 are shown. Bar, expected substitutions per site.

The topology of the Bayesian phylogenetic tree based on 53 core gene sequences (Fig. 1) and the topology of the tree based on genome sequences (Fig. S2) confirm that T1293^T^ is placed with strains AFS055582, AFS073063 and P30BS-XVII in a highly supported cluster distinct from named *Phyllobacterium* species with the type strains of *P. myrsinacearum* and *P. calauticae* as closest relatives. The two trees further show that novel strain T1018 is placed in a highly supported lineage together with the type strain of ‘*P. pellucidum*’ as the closest relative.

Average nucleotide identity (ANI) and digital DNA–DNA hybridization (dDDH) were employed as indices of overall genomic relatedness [20] to facilitate bacterial species delineation. ANI values were calculated using FastANI [24], and the established ANI threshold of 95–96% was employed for species circumscription [20]. Overall genomic relatedness values based on dDDH were calculated using algorithms implemented in the web-based TYGS, and the established dDDH threshold of 70% was used to delineate species boundaries [22]. ANI and dDDH values for pair-wise comparisons of the genome sequence of T1293^T^ with genome sequences of reference strains (AFS055582, AFS073063 and P30BS-XVII and species type strains of closest relatives) are presented in Table 2. The highest ANI and dDDH values were for comparisons of T1293^T^ with type strains of *P. calauticae* (ANI, 84.4%; dDDH, 27%) and *P. myrsinacearum* (ANI, 84.1%; dDDH, 26.5%). These values are well below the accepted thresholds for species delineation and confirm that T1293^T^ represents a novel species within the genus *Phyllobacterium*. Values for genome sequences of strains AFS055582, AFS073063 and P30BS-XVII compared with T1293^T^ were above the respective cutoff values of 95–96% (ANI) and 70% (dDDH) for species circumscription, confirming that these strains belong to the same species cluster as T1293^T^.

**Table 2.** ANI and dDDH values for pair-wise comparisons of genome sequences of *Phyllobacterium meliloti* sp. nov. T1293^T^ (accession nos. CP088273–CP088276) with unclassified strains P30BS-XVII, AFS055582 and AFS073063 (retrieved from NCBI database searches) and species type strains of closest relatives

Reference strain (sequence accession no.)	T1293^T^	
	FastANI	dDDH % (CI)*
*Phyllobacterium* sp. P30BS-XVII (JACGXO01)	98.5	87.1 (84.5–89.3)
*Phyllobacterium* sp. AFS055582 (UEIC01)	98.5	86.7 (84.1–88.9)
*Phyllobacterium* sp. AFS073063 (UCEP01)	98.4	86.7 (84.0–88.9)
*P. calauticae* R2-JL^T^ (JAGENB01)	84.3	27.0 (24.7–29.5)
*P. myrsinacearum* LMG 2t2^T^ (SHLH01)	84.0	26.5 (24.2–29.0)
*Phyllobacterium zundukense* Tri-48^T^ (CP017940-CP017945)	80.0	21.0 (18.8–23.4)
*Phyllobacterium sophorae* CCBAU 03422^T^ (PGGM01)	79.6	20.4 (18.1–22.8)
*Phyllobacterium bourgognense* 31-25a^T^ (QPJM01)	79.5	20.4 (18.2–22.8)
*Phyllobacterium brassicacearum* STM 196^T^ (PGGO01)	79.4	20.3 (18.1–22.7)

*dDDH values based on Genome blast Distance Phylogeny formula 4 implemented in the TYGS.

ANI and dDDH values for the comparison of T1018 with the closely related type strain of ‘*P. pellucidum*’ (98.2% and 84.1%) were above the respective cutoff values of 95–96 and 70% for species circumscription indicating that novel strain T1018 is a member of the species ‘*P. pellucidum*’.

Further genome analyses were carried out using Geneious Prime 2023.0.4 software (www.geneious.com). Based on these analyses, type III secretion system (T3SS) and type VI secretion system (T6SS) genes, implicated in plant–microbe and microbe–microbe interactions [25,27], were detected, respectively, in plasmids pT1293c and pT1293b of novel strain T1293^T^. In contrast, T3SS and T6SS genes were not detected in the genome of ‘*P. pellucidum*’ strain T1018 or the species type strain, BT25^T^. Moreover, we did not detect type II and IV secretion system (T2SS and T4SS) genes [28,29], photosystem genes or genes involved in symbiosis [e.g. nodulation (*nod*) and nitrogen fixation (*nif*) genes], in the genomes of strains T1293^T^ and T1018 or ‘*P. pellucidum*’ BT25^T^. The absence of T4SSs on plasmids in the two novel strains indicates that these plasmids are not self-transmissible [23].

Genes in bacteria encoding 16S, 23S and 5S rRNAs are linked together and expressed as a single ribosomal RNA operon (*rrn*) [30]. Many bacterial species possess multiple *rrn* operon copies in their genomes, and these copies may be located either on chromosomes, plasmids or both [23,30,32]. Our data for the genome analysis of novel strains T1293^T^ and T1018 (Table 1) are in line with these reports: strain T1293^T^ was found to possess three *rrn* copies with two copies located on the chromosome and one copy on plasmid, pT1293a (size, 397.619 kb), whereas ‘*P. pellucidum*’ T1018 was found to possess two *rrn* copies, both on the chromosome. While we did not detect any intragenomic variation between multiple *rrn* operon copies in novel strains T1293^T^ or T1018, this type of heterogeneity is not without precedent and has been reported occasionally in different bacterial species [23,30, 33].

Infectious viruses of bacteria (called bacteriophages or phages) are mediators of horizontal gene transfer and, as a consequence, represent an important driver of bacterial diversification and evolution [34]. In this connection, we used web-based software, PHASTEST [35], to detect potential prophage sequences within the genomes of novel strains T1293^T^ and T1018. Two potential intact (as defined by PHASTEST) prophages were detected in the chromosome of T1293^T^: the first located at co-ordinates 940,853–982,445 bp (size: 41.5 kb) and the second located at coordinates 1,899,457–1,915,246 bp (size: 15.7 kb). The 41.5 kb predicted prophage did not have any close homologues at the nucleotide level, with only a short segment of 4,916 nucleotides of the chromosome of *Phyllobacterium* A18/5-2 (accession number CP104966.1) exhibiting 80% identity based on BLASTn database searches. This predicted prophage sequence contains an integrase gene, genes predicted to be involved in phage replication and a complete set of predicted structural genes for phage assembly, so it is very likely to encode a functional phage, which represents a potential founding member of a novel genus within the class *Caudoviricetes* [International Committee on Taxonomy of Viruses (https://ictv.global/taxonomy/)]. Individual genes encoded predicted proteins with the highest similarity to a variety of phages from organisms like *Sinorhizobium meliloti*, *Paracoccus* species and *Burkholderia* species, and the putative prophage sequence is upstream of several tRNA genes, with tRNAs often being the site of phage integration. The second predicted prophage region (15.7 kb) encoded a complete set of structural proteins with several showing most similarity to proteins of *Rhodobacter* phage RcCronus (NCBI reference: NC_042049) and other *Rhodobacter* phages. However, due to the fact that the region is too small to encode a complete phage genome, and the absence of genes encoding integrases, DNA replication enzymes and other functions, it seems unlikely that this is a functional prophage. In addition, some genes encode proteins that are most similar to those from gene transfer agents (GTAs) [36] of *Rhodobacter* spp. and relatives, raising the possibility that this DNA region encodes a GTA. A potential prophage similar to the 15.7 kb sequence was also detected in the chromosome of ‘*P. pellucidum*’ T1018 (co-ordinates 1,641,578–1,655,055 bp; size, 13.4 kb), but due to its size and lack of some key genes, it might be a GTA-encoding region.

Glyphosate is the most extensively used non-specific herbicide in the world for food production [37]. Its unprecedented scale of use has resulted in negative consequences for human health, non-target plants, fungi and bacteria including the human gut microbiome [37,39]. Consequently, there is considerable interest in deploying bacteria that have the potential to degrade glyphosate to facilitate the bioremediation of contaminated environments [19,37].

Glyphosate specifically inhibits the enzyme 5-enolpyruvylshikimate-3-phosphate synthase (EPSPS), found only in plants, fungi and bacteria and interferes with the synthesis of essential aromatic amino acids in the shikimate pathway [39,40]. EPSPS enzymes have been placed in two classes based on their sensitivity to glyphosate: class I enzymes (found in plants and most Gram-negative bacteria, such as *E. coli*) are naturally sensitive to glyphosate, whereas class II enzymes share less than 30% sequence similarity with class I enzymes, retain their efficiency in the presence of elevated glyphosate concentrations and are found in some naturally occurring glyphosate-tolerant bacteria such as strains of *Agrobacterium* [19,41, 42].

Massot *et al*. [19] reported that unclassified *Phyllobacterium* sp. strain P30BS-XVII was highly resistant to glyphosate. It should be noted that in the present work, we show that strain P30BS-XVII belongs to the same species cluster as novel strain T1293^T^ (Fig. 1).

We analysed the amino acid sequences of EPSPS enzymes in novel strains T1293^T^ (locus tag: LLE53_014710) and ‘*P. pellucidum*’ T1018 (locus tag: LLE51_003070), as well as reference taxa consisting of strains P30BS-XVII (locus tag: FHW17_000551), AFS055582 (not annotated; UEIC01000009 region: 462736–464091), AFS073063 (not annotated; UCEP01000007 region: 489615–490970), *P. myrsinacearum* LMG 2t2^T^ (locus tag: EV654_2109), *P. calauticae* R2-JL^T^ (locus tag: J1W56_14900) [43] and *P. pellucidum* BT25^T^ (locus tag: HQ945_16265) using software implemented in the *EPSPSClass* web server [39]; the results revealed that novel strains and all reference strains possess class II glyphosate-tolerant enzymes, suggesting that this is a general characteristic of *Phyllobacterium* spp.

## Phenotypic characterization

Colonies of strain T1293^T^ are translucent and beige-coloured, spreading with copious gum with diameters of about 1–2 mm after growth on modified YEM agar medium for 3 days at 28 °C. Cells of T1293^T^ are Gram-stain-negative based on the method of [44]. To assess temperature, pH and salt (NaCl) tolerance, bacterial cell suspensions were prepared by adjusting 48 h cultures (grown on modified YEM agar) to ~5×10⁸ cells per millilitre in 0.5% w/v saline. Aliquots (4 µl) of each suspension were spot-inoculated onto triplicate plates of modified YEM agar formulated with the appropriate pH value (pH 5, 6.8 or 10) and NaCl concentration (0.5, 1.0 or 2.0%) or incubated at the desired temperature (10, 28 or 37 °C). The results (Table S1) indicate that strain T1293ᵀ can grow at both pH 5 and pH 10 (with optimal growth at pH 6.8), at temperatures of 10 and 37 °C (with optimal growth at 28 °C) and in the presence of 2% (w/v) NaCl following incubation for 2 days on modified YEM agar; incubation temperature was 28 °C unless otherwise indicated.

Cell morphology was investigated using cells grown in modified YEM broth for 2 days at 28 °C; cells were visualized using transmission (model H-7000; Hitachi) and scanning (model Hitachi SU7000 FESEM) electron microscopes as described previously [23]. The results revealed that cells of T1293^T^ are rod-shaped and possess one or more flagella (Fig. S3).

Further phenotypic characterization using API^®^ strips (API 20NE) and BIOLOG GENIII MicroPlates^™^ was done at BCCM/LMG, Ghent University, Belgium. The results (Table S2) show that novel strain T1293^T^ can be readily differentiated from close relatives *P. calauticae* R2-JL^T^ and *P. myrsinacearum* LMG 2t2^T^, as well as from ‘*P. pellucidum*’ T1018 and ‘*P. pellucidum*’ BT25^T^, based on multiple phenotypic tests.

Antibiotic resistance tests performed on nutrient agar medium in our previous study [7] demonstrated that the novel strain T1293^T^ exhibited resistance to multiple antibiotics, including carbenicillin (>1,000 µg ml^−1^), kanamycin (>100 µg ml^−1^), erythromycin (~100 µg ml^−1^) and tetracycline (>50 µg ml^−1^). These results were supported by our detection of multiple antibiotic resistance genes in the genome of strain T1293^T^ using software implemented in the BV-BRC web-based platform [45]. Antibiotic resistance genes detected included genes encoding enzymes that inactivate beta-lactam antibiotics (e.g. carbenicillin), macrolide antibiotics (e.g. erythromycin), and aminoglycoside antibiotics (e.g. kanamycin and neomycin), as well as genes conferring resistance to tetracycline antibiotics (data not shown).

Notably, aside from tetracycline, novel strain T1018 also exhibited high resistance to the same antibiotics as strain T1293^T^ [7]. Both strains were isolated from a field site with no history of cultivation. Interestingly, the type strain of *Ensifer canadensis* was also recovered from this site and showed comparable levels of antibiotic resistance [7,23]. Additionally, a closely related species, *Ensifer morelensis* [46], that was isolated in Mexico, displayed an almost identical pattern of high-level antibiotic resistance. On the other hand, the species of *Rhizobium* and *Sinorhizobium* that we recovered from the Canadian field site did not exhibit high-level antibiotic resistance [7]. Since all of these species are known to inhabit soil and/or plants, their antibiotic resistance profiles may reflect modification of systems such as efflux pumps, target sites and membrane permeability that promote survival and competitiveness in diverse environments [47].

Analysis of fatty acids was carried out using the Sherlock Microbial Identification System (midi) version 6.0 and the RTSBA6 database as described previously [23] using bacterial cells grown on modified YEM agar for 3 days at 28 °C. The most abundant fatty acids in strain T1293^T^ were C_16 : 0_ (8.3%), C_18 : 1_ ω*7c* 11-methyl (14.2%), C_19 : 0_ cyclo ω8*c* (46.8%) and C_18 : 1_ ω*6c*/_18 : 1_ ω*7c* (summed feature 8) (8.1%), consistent with the profiles of reference strains (Table S3) and with members of the genus *Phyllobacterium* reported previously [48].

Tests were done to assess the ability of novel strains T1293^T^ and T1018 to metabolize glyphosate when it was supplied as the sole source of phosphorus in liquid culture medium; *P. myrsinacearum* LMG 2t2^T^ and *E. coli* K12 derivative (QIAGEN EZ competent cells) were used as EPSPS class II and class I reference strains, respectively. We developed a defined liquid culture medium for these tests: the basal medium had the following composition (g l^−1^) where all ingredients were filter-sterilized unless otherwise indicated with an A (sterilized by autoclaving for 20 min at 15 psi and 121 °C): glucose (2), NH_4_Cl (0.5; A), l-glutamic acid monosodium salt hydrate (0.5), thiamine (0.001), NaCl (0.05; A), MgSO_4_·7H_2_O (0.25 g; A), CaCl_2_·2H_2_O (0.02; A) and FeCl_3_ ⋅ 6H_2_O (0.2). Glyphosate [N-(phosphonomethyl) glycine] ≥95.0% assay (Glentham Life Sciences, UK) as the sole source of phosphorus was added to the basal medium at 1,000 and 2,000 mg l^−1^ (medium BMG). For positive (phosphate) controls, glyphosate was replaced with Na_2_HPO_4_ (0.6; A) and KH_2_PO_4_ (0.3; A); FeCl_3_·6H_2_O was replaced with EDTA ferric sodium salt (0.027) to obviate precipitate formation (medium BMP). The pH of media was adjusted to ~pH 6.5 with 1M KOH.

Inocula were prepared from bacterial cultures grown for 48 h at 29 °C on modified tryptone-yeast extract agar mediumwith the composition (g l^−1^): tryptone (Oxoid, US), 0.5; yeast extract (Oxoid, US), 1.0; calcium chloride dihydrate, 0.1; bacteriological agar no. 1 (Oxoid, US), 15.0. Cells harvested from plates were washed in 0.5% NaCl solution and resuspended to yield about 10^8^ cells ml^−1^; 10 µl aliquots were added to 8 ml volumes of BMG and BMP as appropriate. Bacterial cell suspensions were grown at 29 °C in Falcon^™^ 96-well, non-treated microplates (Corning, USA); 250 µl aliquots of growth medium representing each treatment were added to triplicate wells. To minimize edge effects, wells on outer edges of microplates were filled with sterile water. Bacterial growth was assessed on the basis of OD (595 nm) using a FLUOstar OPTIMA plate reader (BMG LABTECH, Germany) with readings taken at 2.5 h intervals. Bacterial growth curves (Fig. S4) clearly show that novel strains T1293^T^ and ‘*P. pellucidum*’ T1018, as well as reference strain *P. myrsinacearum* LMG 2t2^T^, were able to grow and utilize glyphosate, as the sole source of phosphorus, at the highest level tested (2,000 mg l^−1^); *E. coli* used as a negative control showed, as expected, no growth in the presence of glyphosate. Moreover, these results corroborate the findings of our genome analyses of *Phyllobacterium* strains indicating that they possess class II EPSPS enzymes resistant to glyphosate. Further research is now needed to determine whether novel strains T1293^T^ and T1018, as well as other strains of *Phyllobacterium* spp., have potential as a resource for the bioremediation of glyphosate-contaminated environments.

Plant tests using modified Leonard jars were carried out as described previously [7]. Tests done in this study, as well as in the previous study [7], showed that novel strains T1293^T^ and T1018 did not elicit nodules on the roots of plants of *M. albus* cv. Polara, *Medicago lupulina* (black medic), *Medicago sativa* (alfalfa) or *Macroptilium atropurpureum* (Siratro).

These observations are consistent with the apparent absence of key symbiosis genes (e.g. *nod* and *nif*) in the genomes of *P. meliloti* sp. nov. T1293ᵀ and T1018. Until recently, rhizobia were thought to be the exclusive inhabitants of legume root nodules. It is now known that rhizobia coexist within nodules along with a diverse microbiome of non-nodulating and non-nitrogen-fixing bacteria [49,50**]**. Although *P. meliloti* sp. nov. T1293ᵀ was isolated from a root nodule, other unclassified strains that we identified as belonging to the same novel species (AFS055582 and AFS073063 [18] and P30BS-XVII [19]) were isolated from diverse environments, including soil, an insect and roots of corn (Table 1). Moreover, BT25ᵀ, the type strain of ‘*Phyllobacterium pellucidum*’, was isolated from soil [8], whereas novel strain T1018, also identified as ‘*P. pellucidum*’, was isolated from a root nodule in our earlier work [6]. Collectively, these findings highlight the ecological versatility of these *Phyllobacterium* species, which are not strictly associated with plants but are capable of inhabiting a range of contrasting environments.

## Description of *Phyllobacterium meliloti* sp. nov.

*Phyllobacterium meliloti* (me.li.lo'ti. N.L. gen. n *meliloti*, of the plant genus *Melilotus*).

Colonies are translucent, beige-coloured, spreading with copious gum formation and with diameters of ~1–2 mm after growth on YEM agar medium for 3 days at 28 °C. Cells are Gram-stain-negative, aerobic, non-spore-forming rods with one or more flagella. Bacteria grow at pH 5 and pH 10 (optimal at ~pH 6.8), at temperatures of 10 and 37 °C (optimal at ~28 °C) and in the presence of 2% NaCl, after 2 days on YEM agar medium.

Bacteria are positive for the utilization of 40 carbon sources including d-raffinose, *β*-methyl-d-glucoside, *N*-acetyl-*β*-d-mannosamine, d-saccharic acid, d-galactose, citric acid and d-glucose-6-PO4. Does not utilize 15 carbon sources including glucuronamide, methyl pyruvate, inosine, Tween 40 and d-aspartic acid. Resistant to 12 chemical compounds including minocycline, Niaproof 4 and potassium tellurite but susceptible to sodium bromate. Resistant to >2,000 mg l^−1^ glyphosate.

Positive for urease activity, aesculin hydrolysis and assimilation of mannose, mannitol, *N*-acetyl-glucosamine, maltose, potassium gluconate, malate, trisodium citrate and arabinose.

Bacteria are negative for hydrolysis of gelatin, *ß*-galactosidase activity, arginine dihydrolase, reduction of nitrates to nitrites, reduction of nitrates to nitrogen, indole production and fermentation of glucose. Bacteria do not assimilate capric acid, phenylacetic acid and adipic acid.

Predominant fatty acids (>10%) are C_18 : 1_ ω7c 11-methyl and C_19 : 0_ cyclo ω8c.

The type strain, T1293^T^ (=LMG 32641^T^=HAMBI 3765^T^), was isolated from a root nodule of an *M. albus* (white sweet clover) plant growing in Canada. The type strain possesses key T3SS and T6SS genes but does not possess key nodulation, nitrogen fixation or photosystem genes.

The type strain does not elicit root nodules on plants of *M. albus* (white sweet clover), *M. lupulina* (black medic), *M. sativa* (alfalfa) or *M. atropurpureum* (Siratro).

The DNA G+C content of the type strain is 55.1 mol%, and the genome size is 5.07 Mbp. GenBank/EMBL/DDBJ accession numbers for the complete genome and the 16S rRNA gene sequence of the type strain are, respectively, CP088273–CP088276 and EU928869.

## Supplementary material

10.1099/ijsem.0.006876 Uncited Supplementary Material 1.

## Abbreviations

ANI: average nucleotide identity
BMG: basal growth medium containing glyphosate
BMP: basal growth medium containing phosphate
dDDH: digital DNA–DNA hybridization
EPSPS: 5-enolpyruvylshikimate-3-phosphate synthase
GTAs: gene transfer agents
T3SS: type III secretion system
T4SS: type IV secretion system
T6SS: type VI secretion system
TYGS: Type Strain Genome Server
YEM: yeast extract mannitol

## References

[1] diCenzo GC, Yang Y, Young JPW, Kuzmanović N (2024). Refining the taxonomy of the order *Hyphomicrobiales* (*Rhizobiales*) based on whole genome comparisons of over 130 type strains. Int J Syst Evol Microbiol.

[2] Knösel D (1962). Prüfung von bakterien auf fähigkeit zur sternbildung. Zentralbl Bakteriol Parasitenkd Infektionskr Hyg II Abt.

[3] Parte AC, Sardà Carbasse J, Meier-Kolthoff JP, Reimer LC, Göker M (2020). List of Prokaryotic names with Standing in Nomenclature (LPSN) moves to the DSMZ. Int J Syst Evol Microbiol.

[4] Jiao YS, Yan H, Ji ZJ, Liu YH, Sui XH (2015). *Phyllobacterium sophorae* sp. nov., a symbiotic bacterium isolated from root nodules of *Sophora flavescens*. Int J Syst Evol Microbiol.

[5] Valverde A, Velázquez E, Fernández-Santos F, Vizcaíno N, Rivas R (2005). *Phyllobacterium trifolii* sp. nov., nodulating *Trifolium* and *Lupinus* in Spanish soils. Int J Syst Evol Microbiol.

[6] Bromfield ES, Butler G, Barran LR (2001). Temporal effects on the composition of a population of *Sinorhizobium meliloti* associated with *Medicago sativa* and *Melilotus alba*. Can J Microbiol.

[7] Bromfield ESP, Tambong JT, Cloutier S, Prévost D, Laguerre G (2010). Ensifer, *Phyllobacterium* and *Rhizobium* species occupy nodules of *Medicago sativa* (alfalfa) and *Melilotus alba* (sweet clover) grown at a Canadian site without a history of cultivation. Microbiology.

[8] Park Y, Ten LN, Maeng S, Chang Y, Jung H-Y (2021). *Phyllobacterium pellucidum* sp. nov., isolated from soil. Arch Microbiol.

[9] Bromfield ESP, Cloutier S (2024). *Bradyrhizobium ontarionense* sp. nov., a novel bacterial symbiont isolated from *Aeschynomene indica* (Indian jointvetch), harbours photosynthesis, nitrogen fixation and nitrous oxide (N_2_O) reductase genes. Antonie van Leeuwenhoek.

[10] Kolmogorov M, Yuan J, Lin Y, Pevzner PA (2019). Assembly of long, error-prone reads using repeat graphs. Nat Biotechnol.

[11] Bowers RM, Kyrpides NC, Stepanauskas R, Harmon-Smith M, Doud D (2017). Minimum information about a single amplified genome (MISAG) and a metagenome-assembled genome (MIMAG) of bacteria and archaea. Nat Biotechnol.

[12] Pinto UM, Pappas KM, Winans SC (2012). The ABCs of plasmid replication and segregation. Nat Rev Microbiol.

[13] Edgar RC (2004). MUSCLE: a multiple sequence alignment method with reduced time and space complexity. *BMC Bioinformatics*.

[14] Yu X, Cloutier S, Tambong JT, Bromfield ESP (2014). *Bradyrhizobium ottawaense* sp. nov., a symbiotic nitrogen fixing bacterium from root nodules of soybeans in Canada. Int J Syst Evol Microbiol.

[15] Darriba D, Posada D, Kozlov AM, Stamatakis A, Morel B (2020). Model test-NG: a new and scalable tool for the selection of DNA and protein evolutionary models. Mol Biol Evol.

[16] Miller MA, Pfeiffer W, Schwartz T (2010). 2010 Gateway Computing Environments Workshop (GCE).

[17] Bromfield ESP, Cloutier S, Tambong JT, Tran Thi TV (2017). Soybeans inoculated with root zone soils of Canadian native legumes harbour diverse and novel *Bradyrhizobium* spp. that possess agricultural potential. Syst Appl Microbiol.

[18] Garrido-Oter R, Nakano RT, Dombrowski N, Ma K-W, McHardy AC (2018). Modular traits of the *Rhizobiales* root microbiota and their evolutionary relationship with symbiotic rhizobia. Cell Host Microbe.

[19] Massot F, Gkorezis P, Van Hamme J, Marino D, Trifunovic BS (2020). Isolation, biochemical and genomic characterization of glyphosate tolerant bacteria to perform microbe-assisted phytoremediation. Front Microbiol.

[20] Riesco R, Trujillo ME (2024). Update on the proposed minimal standards for the use of genome data for the taxonomy of prokaryotes. Int J Syst Evol Microbiol.

[21] Jolley KA, Bliss CM, Bennett JS, Bratcher HB, Brehony C (2012). Ribosomal multilocus sequence typing: universal characterization of bacteria from domain to strain. Microbiology.

[22] Meier-Kolthoff JP, Göker M (2019). TYGS is an automated high-throughput platform for state-of-the-art genome-based taxonomy. Nat Commun.

[23] Bromfield ESP, Cloutier S, Hynes MF (2023). *Ensifer canadensis* sp. nov. strain T173^T^ isolated from *Melilotus albus* (sweet clover) in Canada possesses recombinant plasmid pT173b harbouring symbiosis and type IV secretion system genes apparently acquired from *Ensifer medicae*. Front Microbiol.

[24] Jain C, Rodriguez-R LM, Phillippy AM, Konstantinidis KT, Aluru S (2018). High throughput ANI analysis of 90K prokaryotic genomes reveals clear species boundaries. Nat Commun.

[25] Gallique M, Bouteiller M, Merieau A (2017). The type VI secretion system: a dynamic system for bacterial communication?. Front Microbiol.

[26] Dey S, Chakravarty A, Guha Biswas P, De Guzman RN (2019). The type III secretion system needle, tip, and translocon. Protein Sci.

[27] Jiménez-Guerrero I, Medina C, Vinardell JM, Ollero FJ, López-Baena FJ (2022). The rhizobial type 3 secretion system: the Dr. Jekyll and Mr. Hyde in the Rhizobium–legume symbiosis. Int J Mol Sci.

[28] Green ER, Mecsas J (2016). Bacterial secretion systems: an overview. Microbiol Spectr.

[29] Gordils-Valentin L, Ouyang H, Qian L, Hong J, Zhu X (2024). Conjugative type IV secretion systems enable bacterial antagonism that operates independently of plasmid transfer. *Commun Biol*.

[30] Espejo RT, Plaza N (2018). Multiple ribosomal RNA operons in bacteria; their concerted evolution and potential consequences on the rate of evolution of their 16S rRNA. Front Microbiol.

[31] Pei AY, Oberdorf WE, Nossa CW, Agarwal A, Chokshi P (2010). Diversity of 16S rRNA genes within individual prokaryotic genomes. Appl Environ Microbiol.

[32] Anda M, Ohtsubo Y, Okubo T, Sugawara M, Nagata Y (2015). Bacterial clade with the ribosomal RNA operon on a small plasmid rather than the chromosome. Proc Natl Acad Sci USA.

[33] Acinas SG, Marcelino LA, Klepac-Ceraj V, Polz MF (2004). Divergence and redundancy of 16S rRNA sequences in genomes with multiple *rrn* operons. J Bacteriol.

[34] Touchon M, Moura de Sousa JA, Rocha EP (2017). Embracing the enemy: the diversification of microbial gene repertoires by phage-mediated horizontal gene transfer. Curr Opin Microbiol.

[35] Wishart D, Han S, Saha S, Oler E, Peters H (2023). PHASTEST: faster than PHASTER, better than PHAST. Nucleic Acids Res.

[36] Lang AS, Westbye AB, Beatty JT (2017). The distribution, evolution, and roles of gene transfer agents in prokaryotic genetic exchange. Annu Rev Virol.

[37] Singh S, Kumar V, Gill JPK, Datta S, Singh S (2020). Herbicide glyphosate: toxicity and microbial degradation. Int J Environ Res Public Health.

[38] Annett R, Habibi HR, Hontela A (2014). Impact of glyphosate and glyphosate-based herbicides on the freshwater environment. J Appl Toxicol.

[39] Leino L, Tall T, Helander M, Saloniemi I, Saikkonen K (2021). Classification of the glyphosate target enzyme (5-enolpyruvylshikimate-3-phosphate synthase) for assessing sensitivity of organisms to the herbicide. J Hazard Mater.

[40] Funke T, Han H, Healy-Fried ML, Fischer M, Schönbrunn E (2006). Molecular basis for the herbicide resistance of Roundup Ready crops. Proc Natl Acad Sci USA.

[41] Pollegioni L, Schonbrunn E, Siehl D (2011). Molecular basis of glyphosate resistance-different approaches through protein engineering. FEBS J.

[42] Yi S, Cui Y, Zhao Y, Liu Z, Lin Y (2016). A novel naturally occurring class I 5-enolpyruvylshikimate-3-phosphate synthase from *Janibacter* sp. confers high glyphosate tolerance to rice. Sci Rep.

[43] Lustermans JJM, Bjerg JJ, Schramm A, Marshall IPG (2021). *Phyllobacterium calauticae* sp. nov. isolated from a microaerophilic veil transversed by cable bacteria in freshwater sediment. Antonie van Leeuwenhoek.

[44] Buck JD (1982). Nonstaining (KOH) method for determination of gram reactions of marine bacteria. Appl Environ Microbiol.

[45] Olson RD, Assaf R, Brettin T, Conrad N, Cucinell C (2023). Introducing the Bacterial and Viral Bioinformatics Resource Center (BV-BRC): a resource combining PATRIC, IRD and ViPR. Nucleic Acids Research.

[46] Wang ET, Tan ZY, Willems A, Fernández-López M, Reinhold-Hurek B (2002). *Sinorhizobium morelense* sp. nov., a *Leucaena leucocephala*-associated bacterium that is highly resistant to multiple antibiotics. Int J Syst Evol Microbiol.

[47] Nesme J, Simonet P (2015). The soil resistome: a critical review on antibiotic resistance origins, ecology and dissemination potential in telluric bacteria. Environ Microbiol.

[48] Flores-Félix J-D, Carro L, Velázquez E, Valverde Á, Cerda-Castillo E (2013). *Phyllobacterium endophyticum* sp. nov., isolated from nodules of *Phaseolus vulgaris*. Int J Syst Evol Microbiol.

[49] Martínez-Hidalgo P, Hirsch AM (2017). The nodule microbiome: N2-fixing rhizobia do not live alone. Phytobiomes J.

[50] Hnini M, Aurag J (2024). Prevalence, diversity and applications potential of nodules endophytic bacteria: a systematic review. Front Microbiol.

