# Supplementary material for: Characterization of Phyllobacterium spp. isolated from root nodules of Melilotus albus (white sweet clover) grown in Canada and description of Phyllobacterium meliloti sp. nov
Source: Int J Syst Evol Microbiol. 2025 Aug 7;75(8):006876. doi: 10.1099/ijsem.0.006876 (PMC12451606; doi:10.1099/ijsem.0.006876)
Supplement: Uncited Supplementary Material 1. [file ijsem-75-06876-s001.pdf]

Supplementary Material

Characterization of *Phyllobacterium* spp. isolated from root-nodules of *Melilotus albus* (white sweet clover) grown in Canada and description of *Phyllobacterium meliloti* sp. nov.

Eden S. P. Bromfield*, Sylvie Cloutier and Michael F. Hynes

***Correspondence:** Eden S. P. Bromfield, eden.bromfield@agr.gc.ca

Supplementary Table S1. Growth characteristics of *Phyllobacterium* strains: **1**. *P. meliloti* sp. nov. T1293^T, **2.** *P. myrsinacearum* LMG 2t2^T, **3**. *P. calauticae* R2-JL^T, **4.** '*P. pellucidum'* T1018, and, **5**. '*P. pellucidum'* BT25^T.

**Characteristic**	**1**	**2**	**3**	**4**	**5**
Growth on YEM agar medium*					
10 °C	w	w	w	-	-
28 °C	+	+	+	+	+
37 °C	w	w	w	w	w
pH 5	+	+	+	+	+
pH 6.8	+	+	+	+	+
pH 10	+	+	+	+	w
0.5% NaCl	+	+	+	+	+
1% NaCl	+	+	+	+	+
2% NaCl	+	+	w	-	w

* Positive, +; weak, w ; negative, −.

Values are based on three replicates after 48hrs.

All data were generated in this study.

Supplementary Table S2. Phenotypic characteristics of *Phyllobacterium* strains: **1**. *P. meliloti* sp. nov., T1293^T, **2.** *P. calauticae* R2-JL^T, **3.** *P. myrsinacearum* LMG 2t2^T, **4.** *'P. pellucidum'* T1018, and, **5**. *'P. pellucidum'* BT25^T.

**Characteristic**	**1**	**2**	**3**	**4**	**5**	**Characteristic**	**1**	**2**	**3**	**4**	**5**
						C-source utilization (Biolog)					
Dextrin	-	-	w	-	-	Glycyl-L-Proline	+	w	+	w	w
D-Maltose	+	+	+	-	-	L-Alanine	+	+	+	-	-
D-Trehalose	+	+	+	-	-	L-Arginine	w	w	+	w	w
D-Cellobiose	w	+	+	w	w	L-Aspartic Acid	+	+	+	+	+
Gentiobiose	+	+	+	w	w	L-Glutamic Acid	+	+	+	+	+
Sucrose	+	+	+	-	-	L-Histidine	+	w	+	+	+
D-Turanose	+	+	+	w	w	L-Pyroglutamic Acid	w	w	w	-	-
Stachyose	-	-	-	-	-	L-Serine	+	w	+	-	-
D-Raffinose	+	w	-	-	-	Pectin	w	w	+	-	-
α-D-Lactose	-	-	-	-	-	D-Galacturonic Acid	w	+	+	w	w
D-Melibiose	+	+	-	-	-	L-Galactonic Acid Lactone	w	+	+	w	w
β-Methyl-DGlucoside	+	w	w	-	-	D-Gluconic Acid	+	+	+	-	w
D-Salicin	-	-	-	-	-	D-Glucuronic Acid	w	+	+	+	w
N-Acetyl-DGlucosamine	+	+	+	+	+	Glucuronamide	-	+	+	w	w
N-Acetyl-β-DMannosamine	+	-	+	+	+	Mucic Acid	-	-	-	-	-
N-Acetyl-DGalactosamine	+	+	+	+	+	Quinic Acid	-	-	-	-	-
N-Acetyl Neuraminic Acid	-	-	+	-	-	D-Saccharic Acid	+	-	-	-	-
α-D-Glucose	w	+	+	w	w	p-HydroxyPhenylacetic Acid	-	-	-	-	-
D-Mannose	w	+	+	w	w	Methyl Pyruvate	-	w	+	-	-
D-Fructose	+	+	+	+	+	D-Lactic Acid Methyl Ester	w	w	+	w	w
D-Galactose	+	+	+	w	w	L-Lactic Acid	+	+	+	w	w
3-Methyl Glucose	-	-	-	-	-	Citric Acid	+	+	+	-	-
D-Fucose	+	+	+	+	+	α-Keto-Glutaric Acid	w	w	+	w	w
L-Fucose	+	+	+	+	+	D-Malic Acid	+	+	+	+	+
L-Rhamnose	+	+	+	w	w	L-Malic Acid	+	+	+	+	+
Inosine	-	-	-	-	-	Bromo-Succinic Acid	+	w	+	+	+
D-Sorbitol	+	+	+	+	+	Tween 40	-	-	w	-	-
D-Mannitol	+	+	+	+	+	γ-Amino-Butryric Acid	w	w	+	w	w
D-Arabitol	+	+	+	+	+	α-HydroxyButyric Acid	w	w	w	-	-
myo-Inositol	+	+	+	+	+	β-Hydroxy-D,LButyric Acid	+	+	+	+	+
Glycerol	+	+	+	w	w	α-Keto-Butyric Acid	+	w	w	+	+
D-Glucose- 6-PO4	+	-	-	-	-	Acetoacetic Acid	w	w	w	w	w
D-Fructose- 6-PO4	w	w	w	-	-	Propionic Acid	+	w	+	+	+
D-Aspartic Acid	-	-	-	-	-	Acetic Acid	+	+	+	+	+
Gelatin	-	-	-	-	-	Formic Acid	+	+	+	+	+
						Chemical Sensitivity (Biolog)					
1% Sodium Lactate	+	+	+	-	w	Vancomycin	+	+	+	w	w
Fusidic Acid	w	+	+	w	-	Tetrazolium Violet	+	+	+	+	+
D-Serine	w	w	-	-	-	Tetrazolium Blue	w	+	+	w	+
Troleandomycin	+	w	w	+	+	Nalidixic Acid	+	+	w	w	w
Rifamycin SV	+	+	+	+	+	Lithium Chloride	w	w	w	w	w
Minocycline	+	-	w	-	-	Potassium Tellurite	+	+	-	+	+
Lincomycin	+	+	+	+	+	Aztreonam	+	+	+	+	+
Guanidine HCl	w	w	w	-	w	Sodium Butyrate	+	w	w	w	w
Niaproof 4	+	-	-	+	+	Sodium Bromate	-	w	w	-	-
						Metabolism/Assimilation (API 20NE)					
Reduction of nitrates to nitrites	-	-	-	-	-	Assimilation of mannose	+	+	+	+	+
Reduction of nitrates to nitrogen	-	-	-	-	-	Assimilation of mannitol	+	+	+	+	+
Indol production	-	-	-	-	-	Assimilation of N-acetyl-glucosamine	+	+	+	+	+
Fermentation of glucose	-	-	-	-	-	Assimilation of maltose	+	+	+	-	-
Arginine dihydrolase	-	-	-	-	-	Assimilation of potassium gluconate	+	w	w	-	-
Urease activity	+	+	+	+	+	Assimilation of capric acid	-	-	-	-	-
Hydrolysis of esculin	+	+	+	w	w	Assimilation of adipic acid	-	-	-	-	-
Hydrolysis of gelatin	-	-	-	-	-	Assimilation of malate	+	+	+	+	+
ß-galactosidase activity	-	+	-	-	-	Assimilation of trisodium citrate	+	+	+	-	-
Assimilation of glucose	+	+	+	+	+	Assimilation of phenylacetic acid	-	-	-	-	-
Assimilation of arabinose	w	w	w	w	w						

BIOLOG GEN III MicroPlates and API 20NE (48h incubation at 28 ºC): +, Positive; w, weak; −, negative; v, variable.
All data were generated in this study.

Supplementary Table S3. Fatty acid profiles of *Phyllobacterium* strains: **1**. *P. meliloti* sp. nov., T1293^T, **2.** *P. calauticae* R2-JL^T, **3.** *P. myrsinacearum* LMG 2t2^T, **4.** *'P. pellucidum'* T1018, and, **5**. '*P. pellucidum'* BT25^T.

Fatty Acid	1	2	3	4	5
$C_{13:1}$ at 12-13	-	-	0.2	-	-
$C_{14:0}$	-	-	0.2	0.4	0.62
$C_{15:0}$ 3OH	0.4	-	0.3	-	-
$C_{16:0}$	8.3	6.3	8.0	13.9	17.3
$C_{16:0}$ N alcohol	-	-	-	-	0.6
$C_{16:0}$ 2OH	0.5	-	0.2	-	-
$C_{16:0}$ 3OH	7.1	5.0	5.6	0.8	1.1
$C_{16:1}\omega11c$	0.4	-	0.4	-	-
$C_{17:0}$ cyclo	1.8	1.7	2.2	1.8	2.6
$C_{17:0}$ 3OH	0.3	-	0.2	-	-
$C_{17:1}\omega8c$	0.5	-	0.3	0.4	-
$C_{17:0}$	0.3	-	0.2	0.7	1.4
$C_{18:0}$	1.5	1.2	1.3	1.4	1.3
$C_{18:0}$ iso	0.3	-	0.6	-	-
$C_{18:1}$ 2OH	4.9	-	2.0	-	-
$C_{18:0}$ 2OH	1.0	-	-	-	-
$C_{18:0}$ 3OH	-	1.5	1.6	-	-
$C_{18:1}\omega9c$	-	-	0.7	-	-
$C_{18:1}\omega7c$11-methyl	14.2	7.6	6.3	17.3	14.2
$C_{18:3}\omega6c$(6,9,12)	-	-	-	-	1.6
$C_{19:0}$	0.6	-	-	-	-
$C_{19:0}$ cyclo $\omega8c$	46.8	63.8	56.9	35.5	32.5
$C_{20:0}$	-	-	-	-	0.8
$C_{20:1}$ $\omega7c$	0.4	-	0.4	-	-
$C_{20:2}$ $\omega6,9c$	-	2.2	1.2	1.5	3.2
Summed Feature 2*	1.8	2.0	1.7	0.6	2.0
Summed Feature 3*	0.3	-	0.5	1.8	1.4
Summed Feature 5*	-	0.6	-	0.4	0.9
Summed Feature 7*	0.5	1.0	0.2	1.7	1.5
Summed Feature 8*	8.1	7.2	8.9	21.9	17.1

* Summed Features are fatty acids that cannot be resolved reliably from another fatty acid using the chromatographic conditions chosen. The MIDI system groups these fatty acids together as one feature with a single percentage of the total. Summed Feature 2, $C_{12:0}$ aldehyde/?; Summed Feature 3, $C_{16:1}\omega6c$/$C_{16:1}\omega7c$; Summed Feature 5, $C_{18:0}$ ante/$C_{18:2}\omega6,9c$; Summed Feature 7, $C_{19:1}\omega7c$/$C_{19:1}\omega6c$; Summed Feature 8, $C_{18:1}\omega6c$/$C_{18:1}\omega7c$.

-, not detected.

All data were generated in this study.

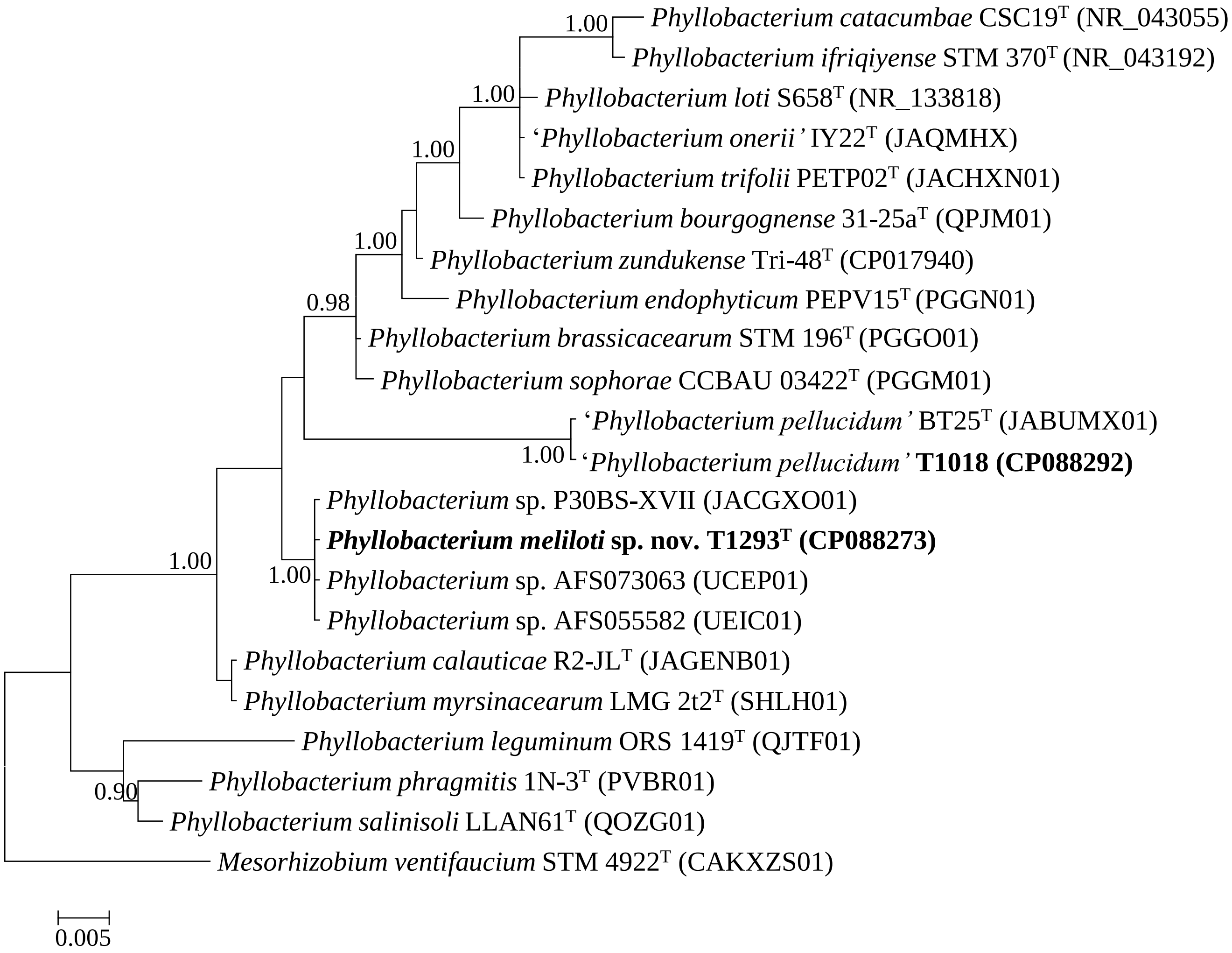

Supplementary Figure S1. Bayesian phylogenetic tree of 16S rRNA gene sequences (1364 positions) for *Phyllobacterium meliloti* sp. nov. strain T1293^T and reference taxa (GTR + G + I substitution model). NCBI sequence accession numbers are given in parentheses. Only posterior probabilities ≥ 0.9 are shown. *Mesorhizobium ventifaucium* STM 4922^T was used as the outgroup. Scale bar represents expected number of substitutions per site.

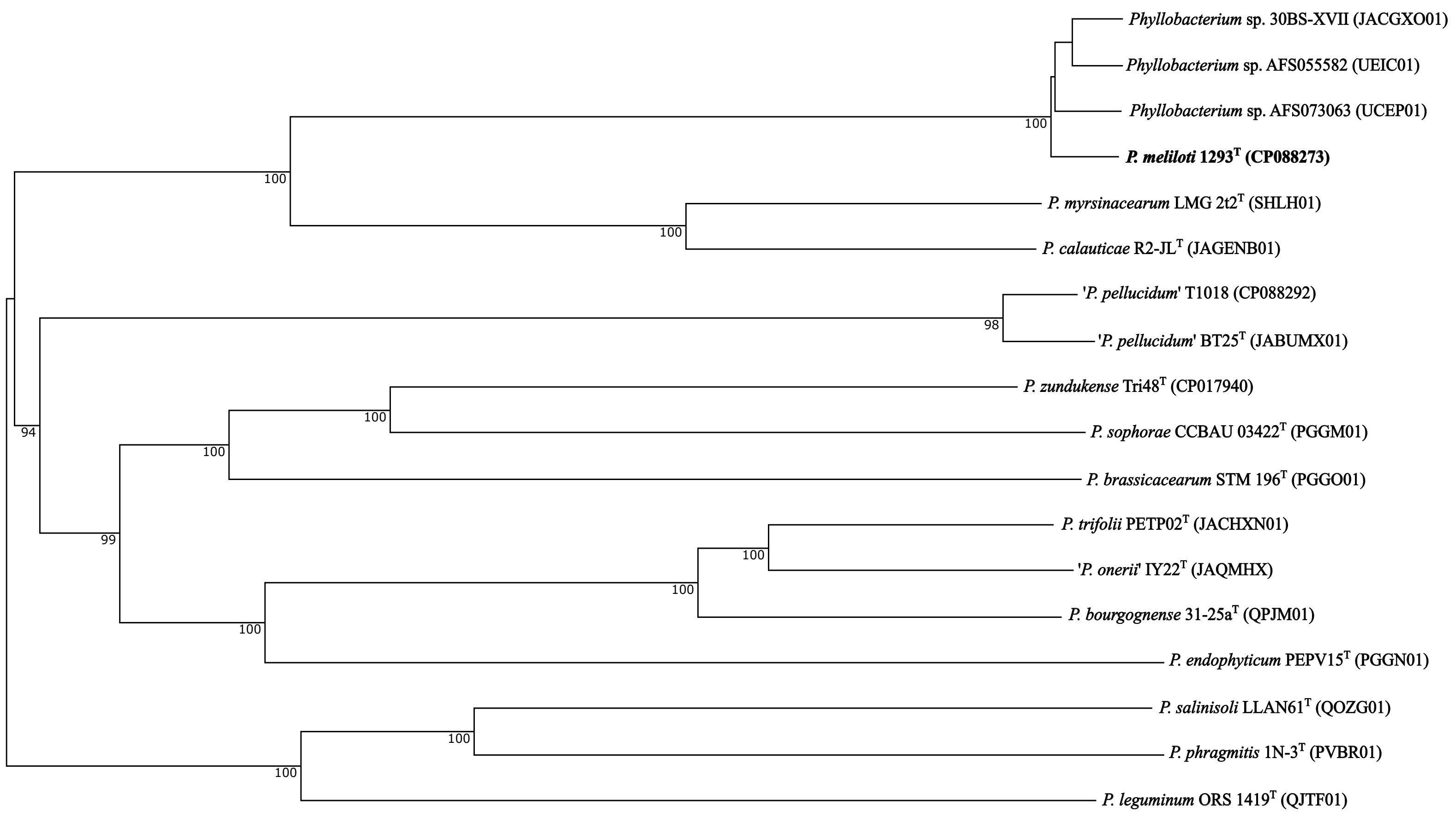

Supplementary Figure S2. Phylogenomic tree based on TYGS implementation (Meier-Kolthoff and Göker, 2019) showing *Phyllobacterium meliloti* sp. nov. T1293^{T} and reference taxa of the genus *Phyllobacterium*. The tree was inferred with FastME 2.1.6.1 from Genome Blast Distance Phylogeny (GBDP) distances calculated from genome sequences. Branch lengths are scaled according to GBDP distance formula d5. The numbers above branches represent GBDP pseudo-bootstrap support values from 100 replications.

A

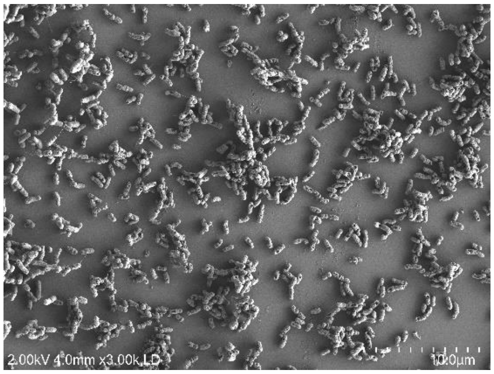

B

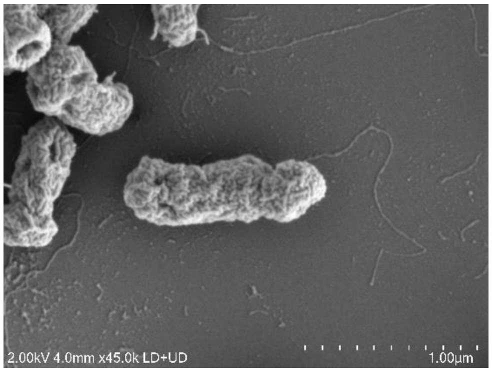

C

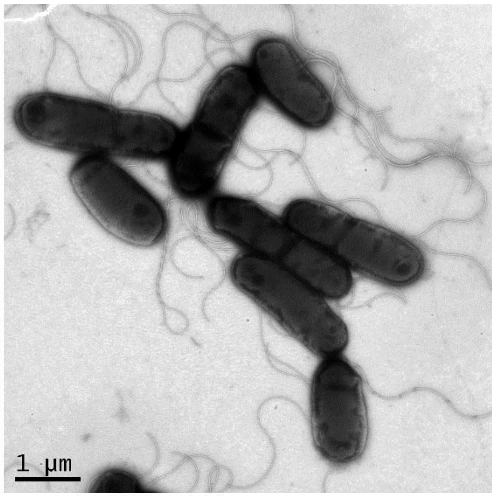

Supplementary Figure S3. Scanning (A and B) and transmission (C) electron microscope images showing morphological features of cells of *Phyllobacterium meliloti* T1293^{T}.

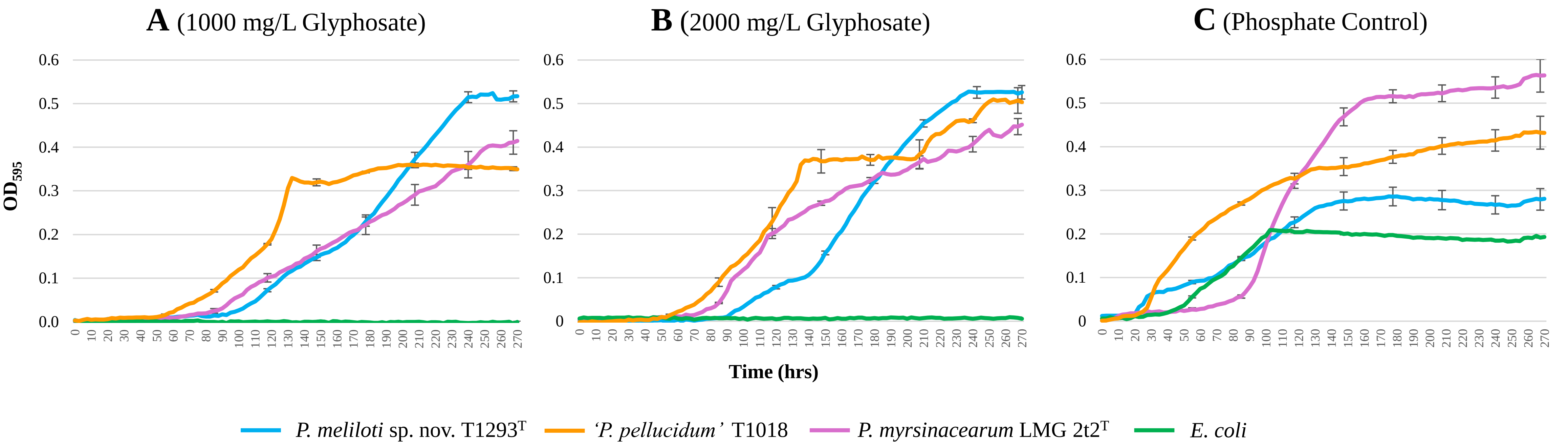

Supplementary Figure S4. Growth (OD 595nm) of *Phyllobacterium meliloti* sp. nov. T1293^{T} and '*P. pellucidum'* T1018 in defined growth medium containing glyphosate as the sole phosphorus source at 1000 mg/L (A) and 2000 mg/L (B). A positive control (C) consisted of defined medium with phosphate substituted for glyphosate. No growth was observed in the negative control in which no phosphorus source was provided. *P. myrsinacearum* LMG 2t2^{T} and *E. coli* were used as glyphosate resistant and sensitive reference strains, respectively. OD readings were taken at 2.5 hour intervals. Values represent means of three replicates; error bars indicate standard errors (SE).
